# Same disease, different outcomes: a retrospective cohort study of COVID-19–associated AKI across Brazil’s dual-tiered healthcare system

**DOI:** 10.1590/2175-8239-JBN-2025-0055en

**Published:** 2025-12-12

**Authors:** Natália Piazzi de Faria, José Hermógenes Rocco Suassuna, Carlos Augusto Moreira de Sousa, Sergio Miranda Freire, Conrado Lysandro R. Gomes

**Affiliations:** 1 Universidade do Estado do Rio de Janeiro, Hospital Universitário Pedro Ernesto, Faculdade de Ciências Médicas, Unidade Docente Assistencial de Nefrologia, Rio de Janeiro, RJ, Brazil.; 2 Kidney Assistance Ltda., Rio de Janeiro, RJ, Brazil.; 3 Complexo Médico Américas, Rio de Janeiro, RJ, Brazil.; 4 Fundação Oswaldo Cruz, Instituto Nacional de Saúde da Mulher, da Criança e do Adolescente Fernandes Figueira, Rio de Janeiro, RJ, Brazil.; 5 Universidade do Estado do Rio de Janeiro, Faculdade de Ciências Médicas, Departamento de Informação, Tecnologias e Educação em Saúde, Rio de Janeiro, RJ, Brazil.

**Keywords:** Acute Kidney Injury, COVID-19, Healthcare Disparities, Mortality, Resource Allocation.

## Abstract

**Introduction:**

Acute kidney injury (AKI) is a critical complication of COVID-19, yet disparities in outcomes between public and private healthcare systems remain underexplored. Brazil’s two-tiered healthcare system offers a unique setting to evaluate how resource allocation impacts AKI outcomes. This study compares AKI epidemiology in COVID-19 patients treated in two Brazilian hospitals, one publicly governed and the other of privately governed.

**Methods:**

This retrospective cohort study analyzed 2,333 ICU patients with RT-PCR–confirmed COVID-19 (public 1,041; private 1,292, March 2020–April 2022). AKI was defined per KDIGO criteria and recovery was classified using ADQI guidelines. Multivariate logistic regression and Cox models were used to assess predictors of AKI incidence and mortality. To account for competing risk of in-hospital death, a Fine-Gray model was used to evaluate renal recovery.

**Results:**

AKI incidence was high in both settings (private, 80.4%; public, 78.8%). Despite comparable baseline characteristics, public hospital patients had significantly higher mortality rates (46.7% vs. 31.3%, p < 0.001). After adjusting for confounders, public hospital admission remained an independent predictor of AKI incidence (OR 1.279, 95% CI 1.012 – 1.620) and mortality (HR 1.675, 95% CI 1.435–1.956). While crude recovery rates appeared higher in public hospitals, competing risk analysis revealed significantly lower recovery probability (SHR 0.650, 95% CI 0.554–0.762).

**Discussion:**

Despite comparable AKI incidence, public hospital patients had higher mortality and lower renal recovery, likely reflecting resource disparities. These findings underscore the need to address cost-effectiveness and equity between the public and private sectors healthcare systems.

## Introduction

Acute kidney injury (AKI) is a common and severe complication of coronavirus disease 2019 (COVID-19) and is associated with poor short- and long-term outcomes^1,2,3^. Since the pandemic onset, a substantial body of research has highlighted the complexity and high mortality rate of AKI in COVID-19 patients^4,5,6,7,8,9,10^.

The global burden of COVID-19 and its severe impact on kidney function has increased disparities in healthcare access and outcomes, particularly in regions with socioeconomic inequality. A prospective study of AKI incidence across 49 countries revealed higher incidence and mortality rates in low- and low-middle-income settings, highlighting the severe consequences of limited access to healthcare resources^11^. Similarly, a systematic review showed that patients from lower socioeconomic settings experienced worse outcomes due to restricted access to critical care resources^12^, which are essential for managing COVID-19-related AKI.

Despite extensive research, the impact of socioeconomic disparities on AKI in Brazil’s dual public-private health system remains unclear. During the pandemic, data indicated that Brazil’s lower socioeconomic regions and public hospitals performed worse, highlighting inequalities in care^13,14^. However, no comparative data exist on the characteristics and outcomes of private and public hospitals. While previous evidence suggests no significant differences in overall AKI outcomes between public and private hospitals in Brazil^15^, COVID-19 posed substantial challenges, with more severe cases and greater demand for intensive care^4^. These issues highlight the need for deeper insights into how resource disparities impact outcomes and for effective interventions to address these gaps.

This study aimed to compare the incidence, recovery, and mortality of AKI in COVID-19 patients admitted to the intensive care units (ICUs) of a public and a private hospital in Brazil, focusing on how differences in healthcare resources between these two settings influence outcomes.

## Methods

### Study Design

This retrospective cohort study included patients admitted to the ICUs with COVID-19 from a public hospital (Pedro Ernesto University Hospital/UERJ) and a private hospital (Americas Medical Complex) in Rio de Janeiro, Brazil, between March 2020 and April 2022. Because the study was observational and used anonymized data, the ethics committees of the participating institutions approved the study (approval number 85672724.8.0000.5533) and waived the requirement for informed consent. The study adhered to the STROBE protocol (Supplementary Material).

### Study Population and AKI Definitions

All patients aged ≥ 18 years who were admitted to the ICUs during the study were included. COVID-19 cases were defined as those that tested positive on RT-PCR. AKI was defined based on the Kidney Disease Global Outcomes (KDIGO) criteria (Table S1)^16^. Baseline creatinine was considered the lowest value during hospitalization^4,6,16,17,18^. Urine output was not recorded in the Epimed Monitor System (see below) and was therefore not used for AKI classification.

Patients with advanced chronic kidney disease (CKD) undergoing kidney replacement therapy (KRT) and those with fewer than two creatinine measurements were excluded. Only the first hospitalization was considered for patients with multiple admissions.

Renal recovery and acute kidney disease (AKD), defined as unresolved AKI lasting more than seven days, were classified according to the Acute Dialysis Quality Initiative (ADQI) criteria^18^. Crude renal recovery was defined as a final serum creatinine level less than 25% above baseline, consistent with the criteria proposed by Pannu et al.^19^. Recovery was classified based on the time interval: early (<48 h), persistent (48 h to 7 days), and late (AKD, > 7 days)^18,20^. For patients requiring KRT, discontinuation of dialysis at the follow-up indicated recovery. For survivors who did not recover within seven days, the ADQI classification for AKD was applied, including stages 0C and 1–3 according to the ADQI definitions (Table S2)^18^. The estimated glomerular filtration rate was calculated using the CKD-EPI 2021 equation using age, sex, and baseline creatinine.

### Sample Size, Data Collection and Sources

**Figure 1.**
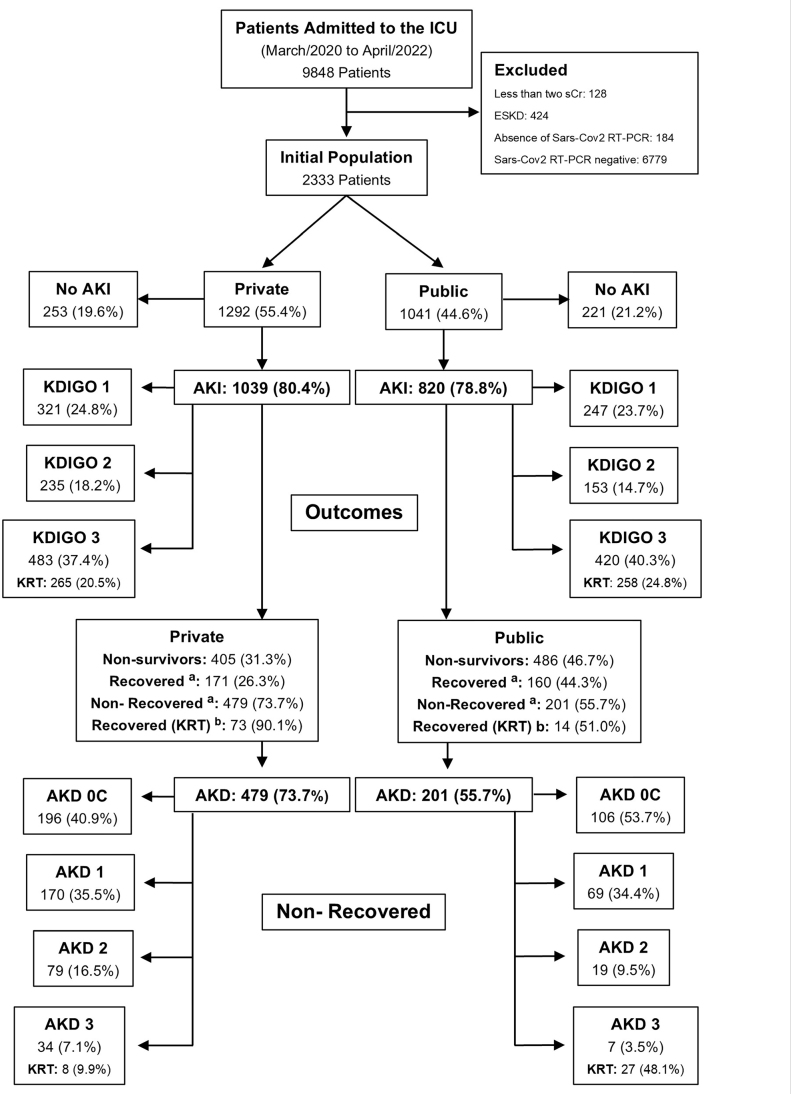
Study flowchart showing patient distribution based on hospital type and AKI (acute kidney injury) occurrence in the ICU (intensive care unit). a. Refers to 1,011 AKI patients with AKI who survived. b. Refers to the 87 patients who underwent KRT and survived. ESKD – end-stage kidney disease, KRT – kidney replacement therapy, sCr – serum creatinine, AKD – acute kidney disease.

Between March 2020 and April 2022, 9,848 patients were admitted to the ICUs of the participating hospitals. After exclusion, the final cohort comprised of 2,333 patients (Figure 1). Data were prospectively collected at ICU admission. The study information was retrieved from the Epimed Monitor System (Epimed Solutions, Rio de Janeiro, Brazil), a structured electronic medical recording system used by ICU case managers^21^ for collection and analysis of data from ICU and ward patients. After registration, patients were followed up prospectively until discharge or inhospital death. The database was anonymized.

Data included demographics, ICU information, comorbidities, Charlson Comorbidity Index (CCI), Modified Frailty Index (MFI), Simplified Acute Physiology Score 3 (SAPS 3), septic shock at admission^22^, use of resources and therapies (vasopressors, non-invasive ventilation, mechanical ventilation (MV), extracorporeal membrane oxygenation (ECMO)), and ICU/hospital mortality. Data entry was timestamped to ensure documentation accuracy. We manually reviewed electronic records to verify the completeness of variables with missing data. However, we excluded race from the multivariate analysis because data were missing for approximately half of the records. Similarly, body mass index (BMI) was excluded due to a high proportion of missing values, as anthropometric data were not systematically collected during the pandemic period.

### Relevant Outcomes

Key outcomes that were compared between public and private hospitals included the incidence and risk factors for AKI, stratified by KDIGO stages, renal recovery in AKI patients, need for KRT, and 30-day in-hospital mortality.

### Statistical Analysis

Data were analyzed using R statistical software (version 4.0.2). Continuous variables are reported as medians with interquartile ranges (IQRs) and were compared using the Mann-Whitney U test. Categorical variables are presented as frequencies and percentages and were compared using the chi-square test. Statistical significance was set at p < 0.05.

Univariate and multivariate logistic regression models were used to identify predictors of AKI incidence and recovery. The covariates included demographic and clinical characteristics, such as age, sex, CCI, MFI, arterial hypertension (AH), diabetes mellitus (DM), CKD, obesity, chronic obstructive pulmonary disease (COPD), cancer, SAPS 3 score, MV, vasopressor use at admission, AKI severity (KDIGO 1–3), and hospital type (public or private).

Multicollinearity was assessed using the variance inflation factor (VIF), and variables with high collinearity were excluded. Because patients with longer hospital stays and greater disease severity are often transferred to public hospitals, we explored the potential selection bias by including an interaction term (hospital type × transfer status) in the regresion model. Adjusted odds ratios (ORs) and 95% confidence intervals (CIs) were calculated. Model selection was based on the Akaike Information Criterion (AIC) and area under the curve (AUC), and goodness of fit was assessed using the Hosmer-Lemeshow test.

Since mortality is a competing event for AKI recovery, a Fine-Gray competing risk model was applied using the *cmprsk* package in R. The time-to-event variable was the length of hospital stay until AKI recovery, with death being treated as a competing event. Patients who remained hospitalized without recovery or death were censored. The predictors included hospital type, MV, and AKI severity (KDIGO stages 1–3, modeled as ordinal variables). Subdistribution hazard ratios (SHRs) with 95% confidence intervals (CIs) were reported, where SHR > 1 indicated a higher probability of recovery.

Kaplan-Meier survival curves were used to estimate 30-day mortality by hospital type, and differences were assessed using the log-rank test. The primary event was in-hospital mortality, with followup beginning at ICU admission and ending with patient death or censoring. Patients who recovered or developed AKD were censored. Univariate Cox proportional hazard models were used to identify predictors of survival, followed by a multivariate Cox model for variables with p < 0.1 in univariate analysis. High multicollinearity was addressed using VIF. Backward selection based on the AIC was applied to refine the final model. The final Cox model included hospital type, severe AKI (KDIGO stage 2/3), MV at admission, and age, which was stratified by transfer status to account for potential admission pathway biases. Model performance was assessed using the likelihood ratio test and concordance index. Results were expressed as hazard ratios (HRs) with 95% CI.

## Results

A total of 9,848 patients were admitted to the ICUs of participating hospitals, with a final cohort of 2,333 COVID-19 patients with positive RT-PCR results being analyzed after exclusion. Of those 1,292 (55.4%) were admitted to the private hospital and 1,041 (44.6%) to the public hospital. Figure 1 depicts the study flowchart with AKI incidence, recovery patterns, and mortality. The incidence of AKI was high, affecting 79.7% of patients, with no significant difference between private and public hospitals (80.4% vs. 78.8%, p = 0.315).

### Demographics, Hospital Admission, Comorbidities, And Clinical Severity

The median age was 64 years, with no significant difference between hospitals (65 vs. 63 years, p = 0.082). A greater percentage of patients was white in the private hospital compared with to the public hospital (74.4% vs. 42.0%, p < 0.001) (Table 1). The median ICU stay was longer in the public hospital (10 vs. nine days, p = 0.002), but the total hospital stay did not differ significantly (p = 0.073). The comorbidity profiles were similar between the groups, with a higher median CCI in the public hospital (p = 0.010). Private hospital patients had higher SAPS-3 scores (median, 49 vs. 46; p < 0.001) and a higher incidence of septic shock at admission (38.8% vs. 25.3%; p < 0.001). Use of MV (52.3% vs. 40.9%, p < 0.001), vasopressors (46.2% vs. 34.8%, p < 0.001), and non-invasive ventilation (including high-flow nasal cannula) (50.9% vs. 25.9%, p < 0.001) were more common in the private hospital. Arterial catheterization, blood transfusion, and extracorporeal membrane oxygenation (ECMO) were also higher in the private hospital group (all p < 0.001) (Table 1).

**Table 1:** Demographics, hospital admission, comorbidities, and clinical severity stratified by hospital type

	All	Private	Public	p-value
	2333 (100%)	1292 (55.4%)	1041 (44.6%)	
**Demographic data**
Age (median [IQR])	64.00 [51.00, 73.00]	65.00 [51.00, 74.00]	63.00 [52.00, 72.00]	0.082
Race (%)^a^				<0.001
White	761 (53.1)	364 (74.4)	397 (42.0)	<0.001
Non-white	674 (46.9)	125 (25.6)	549 (58.0)	
Male sex (%)	1419 (60.8)	851 (65.9)	568 (54.6)	<0.001
**Hospital Admission Data**
Days pre-ICU (median [IQR])	1.00 [0.00, 3.00]	1.00 [0.00, 3.00]	0.00 [0.00, 3.00]	<0.001
ICU stay, days (median [IQR])	9.00 [5.00, 16.00]	9.00 [4.00, 16.00]	10.00 [6.00, 16.00]	0.002
Hospital stay, days (median [IQR])	17.00 [9.00, 29.00]	16.00 [9.00, 27.00]	17.00 [10.00, 30.00]	0.073
Hospital admission type (%)				0.034
Clinical	2311 (99.1)	1282 (99.2)	1029 (98.8)	
Elective surgery	18 (0.8)	6 (0.5)	12 (1.2)	
Emergency surgery	4 (0.2)	4 (0.3)	0 (0.0)	
**Comorbidities**
Charlson Index (median [IQR])	1.00 [0.00, 1.00]	0.00 [0.00, 1.00]	1.00 [0.00, 2.00]	0.010
MFI (median [IQR])	1.00 [0.00, 2.00]	1.00 [0.00, 2.00]	1.00 [0.00, 2.00]	0.090
Systemic arterial hypertension (%)	1363 (58.4)	754 (58.4)	609 (58.5)	0.978
Diabetes mellitus (%)	787 (33.7)	455 (35.2)	332 (31.9)	0.100
Congestive heart failure (%)	112 (4.8)	59 (4.6)	53 (5.1)	0.623
Neoplasia (%)	153 (6.6)	75 (5.8)	78 (7.5)	0.120
Chronic obstructive pulmonary disease (%)	142 (6.1)	116 (9.0)	26 (2.5)	<0.001
Chronic kidney disease (%)	89 (3.8)	48 (3.7)	41 (3.9)	0.864
Obesity (%)	103 (4.4)	44 (3.4)	59 (5.7)	0.011
**Clinical Severity and Resource Use**
Septic shock at admission (%)	764 (32.7)	501 (38.8)	263 (25.3)	<0.001
SAPS-3, points (median [IQR])	48.00 [42.00, 55.00]	49.00 [43.00, 57.00]	46.00 [40.00, 54.00]	<0.001
MV at admission (%)	418 (17.9)	195 (15.1)	223 (21.4)	<0.001
MV (%)	1103 (47.3)	676 (52.3)	426 (40.9)	<0.001
Duration of MV(median [IQR])	10.00 [6.00, 19.00]	9.00 [6.00, 19.00]	11.00 [6.00, 19.00]	0.357
Vasopressors at admission (%)	404 (17.3)	194 (15)	210 (20.2)	<0.001
Vasopressors (%)	959 (41.1)	597 (46.2)	362 (34.8)	<0.001
Non-invasive ventilation (%)	927 (39.7)	657 (50.9)	270 (25.9)	<0.001
Tracheostomy (%)	240 (10.3)	192 (14.9)	48 (4.6)	<0.001
Arterial catheter (%)	1521 (65.2)	1003 (77.6)	518 (49.8)	<0.001
Temporary pacemaker (%)	8 (0.3)	2 (0.2)	6 (0.6)	0.169
Total parenteral nutrition (%)	12 (0.5)	10 (0.8)	2 (0.2)	0.097
Blood transfusion (%)	281 (12.0)	215 (16.6)	66 (6.3)	<0.001
Extracorporeal oxygenation (%)	48 (2.1)	46 (3.6)	2 (0.2)	<0.001

Abbreviations – IQR (Interquartile Range), ICU (Intensive Care Unit), MFI (Modified Frailty Index), SAPS-3 (Simplified Acute Physiology Score 3), MV (Mechanical Ventilation).

Notes – a. Data refers to 1,435 patients with available race information.

### AKI Characteristics and Clinical Predictors

Both hospital types showed similar KDIGO stage distributions, the majority being KDIGO stage 3 (37.4% private vs. 40.3% public, p = 0.086) (Table 2). KRT was more common in public hospitals (24.8% vs. 20.5%; p = 0.016). The use of KRT modalities varied significantly: intermittent hemodialysis (IHD) was more frequent in public hospitals (94.6%), whereas prolonged intermittent (PIKRT) and continuous kidney replacement therapy (CKRT) were more common in private hospitals (55.5% and 37.7%, respectively; p < 0.001). The median duration of KRT was longer in private hospitals (11.0 vs. 7.0 days, p < 0.001) (Table 2).

**Table 2:** AKI Characteristics and recovery among hospital types

	All	Private	Public	p-value
	2333 (100%)	1292 (55.4%)	1041 (44.6%)	
**AKI caractheristics**
Creatinine D1 (median [IQR])	1.04 [0.80, 1.40]	1.00 [0.88, 1.28]	1.20 [0.90, 1.70]	<0.001
eGFR, D1 (median [IQR])	73.6 [49.7, 95.2]	81.2 [60.0, 78.1]	60 [37.4, 84.8]	<0.05
AKI (%)	1859 (79.7)	1039 (80.4)	820 (78.8)	0.315
Acute kidney injury, stages (%)				0.086
No AKI	474 (20.3)	253 (19.6)	221 (21.2)	
KDIGO 1	568 (24.3)	321 (24.8)	247 (23.7)	
KDIGO 2	388 (16.6)	235 (18.2)	153 (14.7)	
KDIGO 3	903 (38.7)	483 (37.4)	420 (40.3)	
KRT (%)	523 (22.4)	265 (20.5)	258 (24.8)	0.016
KRT modality				<0.001
IHD	262 (50.1)	18 (6.8)	244 (94.6)	
PIKRT	161 (30.8)	147 (55.5)	14 (5.4)	
CKRT	100 (19.1)	100 (37.7)	0 (0)	
Median duration of KRT (median [IQR])	8.00 [3.00, 17.00]	11.00 [4.00, 20.00]	7.00 [3.00, 14.00]	<0.001
**Recovery (overall and in AKI survivors)**
Recovered, overall (%)^a^	449 (24.2)	224 (21.6)	225 (27.4)	<0.01
Classification of recovery (%)^a^				<0.05
Not recovered	1410 (75.8)	815 (78.4)	595 (72.6)	
Early recovery (< 2 days)	294 (15.8)	146 (14.1)	148 (18.0)	
Persistent AKI (2 to 7 days)	85 (4.6)	45 (4.3)	40 (4.9)	
Late AKI recovery (> 7 days)	70 (3.8)	33 (3.2)	37 (4.5)	
Recovered, survivors (%)^b^	331 (32.7)	171 (26.3)	160 (44.3)	<0.001
Classification of recovery (%)^b^				<0.001
Non-recovered	680 (67.3)	479 (73.7)	201 (55.7)	
Early recovery (< 2 days)	230 (22.7)	119 (18.3)	111 (30.7)	
Persistent AKI (2 to 7 days)	57 (5.6)	32 (4.9)	20 (3.1)	
Late AKI recovery (> 7 days)	44 (4.4)	20 (4.9)	24 (6.6)	
Recovered from KRT^c^	87 (80.6)	73 (90.1)	14 (51.9)	<0.001
**Non-recovered**
AKD (%)^d^	680 (67.3)	479 (73.7)	201 (55.7)	<0.01
KDIGO AKD 0C	302 (44.4)	196 (40.9)	106 (53.7)	
KDIGO AKD 1	239 (35.1)	170 (35.5)	69 (34.3)	
KDIGO AKD 2	98 (14.4)	79 (16.5)	19 (9.5)	
KDIGO AKD 3	41 (6.1)	34 (7.1)	7 (3.5)	
Mortality in AKI (%)	848 (45.6)	389 (37.4)	459 (56)	<0.001
Mortality, overall (%)	891 (38.2)	405 (31.3)	486 (46.7)	<0.001

Abbreviations – IQR (Interquartile Range), eGFR (Estimated Glomerular Filtration Rate), AKI (Acute Kidney Injury), KDIGO (Kidney Disease Improving Global Outcomes), KRT (Kidney Replacement Therapy), IHD (Intermittent Hemodialysis), PIKRT (Prolonged Intermittent Kidney Replacement Therapy), CKRT (Continuous Kidney Replacement Therapy), and AKD (Acute Kidney Disease).

Notes – a. Refers to all 1,859 patients with AKI, considering death as non-recovery.

b. Refers to the 1,011 AKI patients who survived.

c. Refers to the 108 patients who underwent KRT and survived.

d. Refers to the 680 surviving patients who did not recover.

Multivariate logistic regression analysis showed that increased SAPS-3 scores (odds ratio [OR]: 1.049; 95% CI: 1.035–1.065; p < 0.001), MV (OR 9.721, 95% CI, 5.978–16.446; p < 0.001), AH (OR 1.487, 95% CI, 1.175–1.883; p < 0.001), and CKD (OR 4.662, 95% CI, 1.853–15.670; p < 0.01) were significant risk factors for AKI. Public hospital admission was associated with a slight increase in the odds of AKI (OR 1.279, 95% CI, 1.012–1.620; p < 0.05) (Table 3).

**Table 3:** UNI- and multivariate logistic regression for AKI incidence and recovery

	Univariate			Multivariate		
	OR	95% CI	p	OR	95% CI	p
**Logistic regression for acute kidney injury^a^**						
Arterial hypertension	1.804	1.473–2.211	<0.001	1.487	1.175–1.883	<0.001
Chronic kidney disease	5.630	2.334–18.499	<0.001	4.662	1.853–15.670	<0.01
Public hospital	0.903	0.738–1.107	0.326	1.279	1.012–1.620	<0.05
Mechanical ventilation	22.751	15.66–34.69	<0.001	9.721	5.978–16.446	<0.001
SAPS-3 (per point)	1.084	1.071–1.098	<0.001	1.049	1.035–1.065	<0.001
Vasopressors	18.973	12.74–29.65	<0.001	2.650	1.555–4.636	<0.001
**Logistic regression for kidney function recovery^b^**						
AKI KDIGO 1		Reference			Reference	
AKI KDIGO 2	0.196	(0.146–0.261)	<0.001	0.292	(0.214–0.393)	<0.001
AKI KDIGO 3	0.065	(0.048–0.086)	<0.001	0.143	(0.102–0.198)	<0.001
Mechanical Ventilation	0.099	(0.077–0.126)	<0.001	0.258	(0.193–0.341)	<0.001
Public Hospital	1.162	(0.970–1.392)	0.104	1.087	(0.880–1.344)	0.438

Abbreviations – eGFR (Estimated Glomerular Filtration Rate), AKI (Acute Kidney Injury), KDIGO (Kidney Disease Improving Global Outcomes), SAPS-3 (Simplified Acute Physiology Score 3).

Notes – a. Hosmer-Lemeshow: p-value = 0.675; Area under the curve: 0.835 (95% CI: 0.817–0.853);

b. Hosmer-Lemeshow: p-value = 0.9338; Area under the curve: 0.7599 (95% CI: 0.7399–0.7800).

### AKI Recovery and Predictors

The crude AKI recovery rates were higher in the public hospital group (27.4% vs. 21.6%, p < 0.01; survivors: 44.3% vs. 26.3%, p < 0.001). Recovery from KRT was more prevalent in the private hospital (90.1% vs. 51.9%, p < 0.001) (Table 2).

Non-recovered patients in the private hospital were more often male (66.4% vs. 53.2%, p = 0.002). Septic shock and higher SAPS-3 scores were more common in non-recovered private hospital patients (36.3% vs. 12.9%, p < 0.001; SAPS-3: 49.33 vs. 46.43, p = 0.001). MV (53.9% vs. 23.9%, p < 0.001) was more frequent in non-recovered private hospital patients (Table S3).

The adjusted logistic regression showed that public hospital admission was associated with a higher likelihood of AKI recovery, but this was not statistically significant (OR 1.087, 95% CI, 0.880–1.344; p = 0.104). MV (OR 0.258, p < 0.001) and higher AKI stage (KDIGO 2: OR 0.292, p < 0.001; KDIGO 3: OR 0.143, p < 0.001) significantly reduced the probability of recovery (Table 3).

Contrary to the logistic model findings, the Fine-Gray model showed that public hospital admission was associated with a significantly lower recovery probability (SHR 0.650, 95% CI, 0.554–0.762; p < 0.001). MV (SHR 0.222, p < 0.001) and severe AKI (SHR 0.338, p < 0.001 per stage) were strong negative predictors of recovery (Table 4).

**Table 4:** Fine and gray competitive risk model for AKI recovery

	Subdistribution HR	95% CI	p
Hospital type (public vs. private)	0.650	(0.554–0.762)	<0.001
Mechanical ventilation	0.222	(0.174–0.283)	<0.001
Acute Kidney Injury (per stage)	0.338	(0.292–0.391)	<0.001

Abbreviations – HR: Hazards ratio.

### Survival and COX Regression Analysis

**Figure 2.**
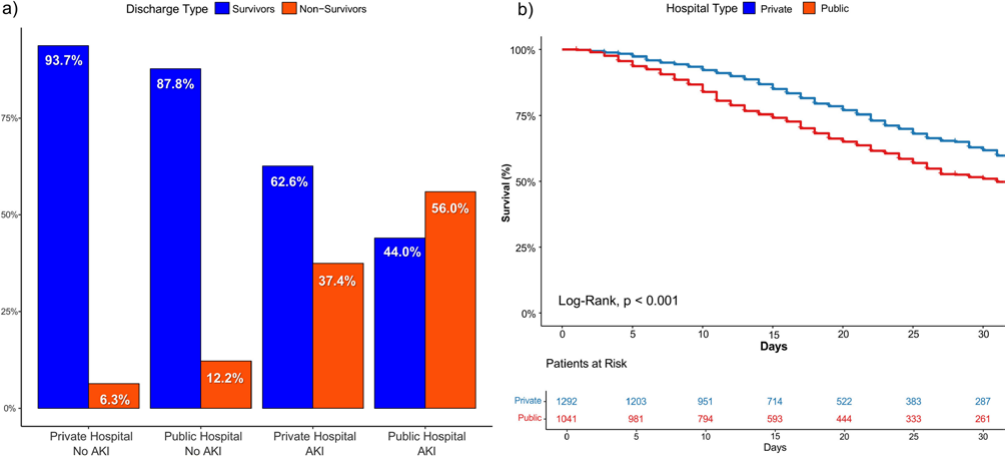
a) Bar chart displaying hospital discharge outcomes (survivors vs. non-survivors) stratified by hospital type and AKI status. b) Kaplan-Meier survival curve comparing ICU patients from public and private hospitals. The log-rank test indicated a significant difference in survival between hospital types (p < 0.001).

The overall mortality rate was higher in the public hospital group (overall: 46.7% vs. 31.3%; in AKI patients, 56% vs. 37.4%, p < 0.001) (Figure 2a). The Kaplan-Meier survival curves showed a lower survival rate in the public hospital (p < 0.001) (Figure 2b).

Non-survivors in the private hospital were older (mean age 70.3 vs. 65.7 years, p < 0.001) and more likely to be male (66.4% vs. 54.7%, p < 0.001). They also had higher rates of comorbidities such as hypertension (70.1% vs. 60.5%, p < 0.001) and DM (41.0% vs. 35.0%, p = 0.001). Clinical severity was greater among non-survivors in private hospitals, as indicated by higher SAPS 3 scores (mean 58.6 vs. 51.5, p < 0.001), incidence of septic shock at admission (60.7% vs. 44.0%, p < 0.001), MV (89.4% vs. 70.4%, p < 0.001), and vasopressor use (81.5% vs. 60.1%, p < 0.001) (Table S4).

In the final Cox proportional hazards model stratified by transfer status, the critical predictors of in-hospital mortality included older age (HR 1.031, 95% CI, 1.026–1.036; p < 0.001), MV (HR 2.548, 95% CI, 2.110–3.076; p < 0.001), AKI severity (HR 1.423, 95% CI, 1.145–1.767; p = 0.001), and public hospital admission (HR 1.675, 95% CI, 1.435–1.956; p < 0.001) (Table 5).

**Table 5:** UNI- and multivariate Cox proportional hazards for in-hospital mortality

	Univariate			Multivariate		
	HR	95% CI	p	HR	95% CI	p
Age	1.029	1.0232–1.035	<0.001	1.031	1.026–1.036	<0.001
AKI KDIGO 2/3	1.388	1.1174–1.725	0.003	1.423	1.145–1.767	0.001
Chalson comorbidity index	1.012	0.9718–1.054	0.568	–	–	–
Diabetes Mellitus	0.91	0.7718–1.074	0.266	–	–	–
Mechanical ventilation	2.408	1.9657–2.949	<0.001	2.548	2.110–3.076	<0.001
Modified frailty index	1.021	0.9546–1.092	0.542	–	–	–
Public hospital	1.966	1.6994–2.275	<0.001	1.675	1.435–1.956	<0.001
Sepsis at admission	1.289	1.0943–1.519	0.002	–	–	–
SAPS-3 (per point)	1.004	0.9967–1.011	0.290	–	–	–

Abbreviations – AKI (Acute Kidney Injury), KDIGO (Kidney Disease Improving Global Outcomes), SAPS-3 (Simplified Acute Physiology Score 3). HR: Hazards ratio.

## Discussion

This study shows the existence of important disparities in the outcomes of COVID-19-associated AKI between public and private hospitals in Brazil. Despite the similar incidence rates of AKI in both hospital settings, our findings revealed significant differences in mortality, resource utilization, and recovery, which can be attributed to variations in healthcare access and quality. These disparities occurred within a broader context of systemic challenges faced by the Brazilian healthcare system during the pandemic, including well-documented difficulties in national coordination and resource distribution^23^.

Consistent with our results, AKI studies during the pandemic showed that COVID-19 patients have not only a higher AKI incidence but also more severe forms, as evidenced by higher rates of stage 3 AKI and a greater need for KRT. Moreover, these patients require more intensive care resources, such as MV, and present with higher disease severity^4,5,6,9,24^. Even after controlling for key variables, the independent influence of COVID-19 status on AKI incidence^4^ suggests that the virus contributes to a more severe AKI phenotype, potentially through direct viral effects, heightened inflammatory responses, or associated systemic complications^1,10,25^. A strong and independent correlation between MV and AKI incidence (OR 9.721, 95% CI 5.978–16.446) and KRT requirement was also noted in multiple COVID-19 cohorts^5,9^, possibly indicating the complex lungkidney crosstalk in AKI pathogenesis^1,10,25^. Although crude AKI incidences did not differ among hospital types, admission to the public hospital persisted as an independent factor for AKI (OR 1.279, 95% CI 1.012–1.620) despite the private hospital’s higher overall disease severity, which was already noted in previous cohorts and possibly linked to lower resource use^11^.

One of the key findings of this study was the higher mortality rate among AKI patients in public hospitals than in private hospitals (46.7% vs. 31.3%, respectively). This stark contrast persisted even after controlling for confounding variables such as age, comorbidities, and clinical severity, suggesting that the differences in outcomes were not solely due to patient health status upon admission. Public hospital admission was identified as an independent predictor of higher mortality, underscoring the impact of healthcare system inequities, which has already been observed in overall COVID-19 mortality in Brazil^13^. These findings also align with global reports highlighting poorer outcomes in healthcare settings with limited resources, which can be exacerbated during public health crises such as the COVID-19 pandemic^11^. Few studies comparing public and private hospitals in Brazil during COVID-19 have reported worse outcomes in public and mixed hospitals^13^. However, these studies did not evaluate differences in AKI incidence or characteristics between hospitals. Previous epidemiological data in Brazil suggest that treatment in public hospitals did not influence survival in dialysis-requiring AKI^15^. However, considering the overall severity of COVID-19 presentation and increased ICU resources required, public hospitals are likely to underperform because of greater constraints than private hospitals. A multinational cohort study in different income settings showed that AKI in COVID-19 patients in low-resource environments had the highest hospital mortality rates. This association persisted even after adjusting for disease severity^11^. Therefore, income and resource availability may directly influence the outcomes of COVID-19 patients. Another report showed that during the peak of COVID-19 severity, hospitals with overwhelmed resources, including those with high ICU occupancy, had higher mortality rates. These findings were most pronounced in regions with limited healthcare resources^26^. Moreover, public hospitals in Brazil often serve a significant proportion of lower socioeconomic populations, which may have delayed access to healthcare, fewer resources, and less comprehensive post-acute care than private hospitals^27^.

In our study, the increased incidence of KRT and frequent use of IHD in public hospitals suggest that these facilities had limited access to more advanced or continuous KRT modalities, which are better tolerated in patients with hemodynamic instability and enable more effective volume control and nutritional support, both of which are relevant in the care of patients with COVID-19^2,25^. Similarly to national studies highlighting healthcare inequalities in Brazil^13,14^, our data revealed substantial differences in the availability and use of advanced resources across hospital types. Private hospital patients were more frequently managed with non-invasive ventilation (including high-flow nasal cannula), ECMO, arterial catheterization, blood transfusions, and tracheostomy. These disparities likely reflect better access to critical care infrastructure and specialized technologies in private settings, which may partially account for the observed differences in mortality and recovery outcomes.

Owing to the high percentage of missing data, race could not be included in our multivariate models. Evidence from high-income countries and Brazil has indicated worse overall outcomes in patients of African descent^12,14^. AKI incidence was also higher in patients of African descent in high-income countries, even after adjusting for socioeconomic factors^4,8^. However, despite the well-known AKI racial differences, comprehensive epidemiological data on AKI in Brazil did not identify race as a significant factor for AKI mortality^15^, and models predicting AKI and KRT in the context of COVID-19 in Brazil consistently did not include race as a covariate^28^.

An initial counterintuitive finding was higher apparent renal recovery from AKI in public hospitals. This could be attributed to more severe disease stage in private hospitals, where patients require more MV and have higher overall severity scores. However, logistic regression does not account for competing events, particularly mortality, which may have introduced survivor bias into recovery rates. To address this, we applied a Fine-Gray competing risk model, which confirmed that public hospital admission was associated with a significantly lower probability of renal recovery (SHR 0.650, 95% CI, 0.554–0.762; p < 0.001). These findings indicate that the higher recovery rates observed in public hospitals using logistic regression were confounded by the higher mortality rates in these patients, as many critically ill individuals did not survive long enough to show renal recovery. Complete recovery from KRT was more prevalent in private hospitals (90.1% vs. 51.9%; p < 0.001). This may reflect differences in illness severity leading to KRT initiation, resource availability, treatment strategies, and availability of skilled healthcare professionals^13^. Private hospitals may have provided more comprehensive care for severe AKI cases, including more intense use of CKRT, which might have resulted in better recovery of kidney function among those requiring KRT^25^. However, this superiority remains debatable^29^. This pattern was further supported by the marked disparity in KRT dependence among patients with stage 3 AKD, with 48.1% in public hospitals versus only 9.9% in private hospitals remaining KRT-dependent. These findings highlight the influence of resource availability and quality of care on kidney recovery trajectories following severe AKI.

The high prevalence of AKD in COVID-19 survivors highlights the long-term renal implications of COVID-19 and the need for long-term follow-up and management strategies^3^. Fisher et al.^4^ described that recovery rates were lower in COVID-19 patients with AKI, with only 42.3% showing kidney recovery compared to 68.5% in non-COVID AKI patients. Emerging data suggest that many patients experience persistent renal impairment after recovery from acute COVID-19^30^. These observations underscore the need for vigilant follow-up and early therapeutic interventions to mitigate the long-term burden of kidney injury in COVID-19 survivors.

This study has several strengths and limitations. The strengths include the large number of patients, prospective data collection, and reflection on real-world care. Limitations include the retrospective design and potential biases from missing racial data, as well as incomplete anthropometric data, which precluded BMI-based analyses. Residual and unmeasured confounders cannot be ruled out, as in any observational study. The study sample was from a specific geographical region, which may limit the generalization to other populations or settings. Another limitation was the absence of longterm follow-up of the survivors. A potential bias was patient referrals from other public units, many already admitted with MV, and critically ill patients, which could have influenced public hospital mortality. However, all models were adjusted for transfer status, indicating that late referral did not significantly affect the observed disparities in outcomes between hospital types.

## Conclusions

This study found that, despite similar AKI incidence, patients with COVID-19 admitted to public hospitals had significantly higher mortality, lower probability of renal recovery after accounting for competing risks, and reduced access to advanced life support technologies such as CKRT and ECMO. These findings emphasize the urgent need to re-evaluate how healthcare resources are allocated across different hospital settings and to consider equity of care in dual healthcare systems in emerging countries. Strengthening critical care capacity and promoting equitable access to advanced therapies remain essential to improving outcomes in resource-constrained settings.

## Data Availability

The dataset analyzed during the current study is available upon reasonable request to the corresponding author.

## Supplementary Material

The following online material is available for this article:

Table S1 Acute kidney injury definitions based on the KDIGO definitions.

Table S2 Acute kidney disease definitions according to ADQI guidelines.

Table S3 Differences in non-recovered patients according to hospital type.

Table S4 Differences between survivors and nonsurvivors, stratified by hospital type.

**STROBE** guidelines checklist.
