# Supplementary material for: Same disease, different outcomes: a retrospective cohort study of COVID-19–associated AKI across Brazil’s dual-tiered healthcare system
Source: J Bras Nefrol. 2025 Dec 12;48(2):e20250055. doi: 10.1590/2175-8239-JBN-2025-0055en (PMC12700444; doi:10.1590/2175-8239-JBN-2025-0055en)
Supplement: Table S1 [file 2175-8239-jbn-48-2-e20250055-suppl1.pdf]

*Braz. J. Nephrol*

https://doi.org/10.1590/2175-8239-JBN-2025-0055en

# Supplementary Material to "Same Disease, Different Outcomes: A Retrospective Cohort Study of COVID-19–Associated AKI Across Brazil's Dual-Tiered Healthcare System"

**TABLE S1** Acute kidney injury definitions based on the KDIGO definitions.

| Stage | Serum creatinine | Urine output |
|---|---|---|
| 1 | 1.5–1.9 times baseline OR Increase by ≥0.3 mg/dl (≥26.5 mmol/l) | <0.5 ml/kg/h for 6–12 hours |
| 2 | 2.0–2.9 times baseline | <0.5 ml/kg/h for >12 hours |
| 3 | ≥3.0 times baseline OR Increase in serum creatinine to ≥4.0 mg/dl (≥353.6 mmol/l) OR Initiation of renal replacement therapy OR, In patients ≤18 years, decrease in eGFR to ≤35 ml/min per 1.73 m^2 | <0.3 ml/kg/h for >24 hours OR Anuria for >12 hours |
