# Supplementary material for: Same disease, different outcomes: a retrospective cohort study of COVID-19–associated AKI across Brazil’s dual-tiered healthcare system
Source: J Bras Nefrol. 2025 Dec 12;48(2):e20250055. doi: 10.1590/2175-8239-JBN-2025-0055en (PMC12700444; doi:10.1590/2175-8239-JBN-2025-0055en)
Supplement: Table S2 [file 2175-8239-jbn-48-2-e20250055-suppl2.pdf]

Braz. J. Nephrol

https://doi.org/10.1590/2175-8239-JBN-2025-0055en

Supplementary Material to “Same Disease, Different Outcomes: A Retrospective Cohort Study of COVID-19–Associated AKI Across Brazil’s Dual-Tiered Healthcare System”

TABLE S2 Acute kidney disease definitions according to ADQI guidelines.

Stage	Definition
Stage 0	
A	Absence of criteria for B or C.
B	Continued evidence of ongoing injury, repair and/or regeneration or indicators of loss of renal glomerular or tubular reserve
C	Serum creatinine level <1.5 times baseline but not back to baseline levels
Stage 1	Serum creatinine level 1.5–1.9 times baseline
Stage 2	Serum creatinine level 2.0–2.9 times baseline
Stage 3	Serum creatinine level ≥3.0 times baseline or increase in serum creatinine to ≥353.6 µmol/l (≥4.0 mg/dl) or ongoing need for renal replacement therapy
