# Supplementary material for: Same disease, different outcomes: a retrospective cohort study of COVID-19–associated AKI across Brazil’s dual-tiered healthcare system
Source: J Bras Nefrol. 2025 Dec 12;48(2):e20250055. doi: 10.1590/2175-8239-JBN-2025-0055en (PMC12700444; doi:10.1590/2175-8239-JBN-2025-0055en)
Supplement: Table S3 [file 2175-8239-jbn-48-2-e20250055-suppl3.pdf]

Braz. J. Nephrol

https://doi.org/10.1590/2175-8239-JBN-2025-0055en

Supplementary Material to "Same Disease, Different Outcomes: A Retrospective Cohort Study of COVID-19–Associated AKI Across Brazil's Dual-Tiered Healthcare System"

TABLE S3 Difference in Non-recovered Patients according to Hospital Type.

	Overall	Private Hospital	Public Hospital	p
n	680	479	201	
Age (mean (SD))	58.14 (15.66)	57.99 (15.37)	58.51 (16.37)	0.691
Sex (%)	425 (62.5)	318 (66.4)	107 (53.2)	0.002
Ethnicity				<0.001
White	14 (2.1)	6 (1.3)	8 (4.0)	
Non-White	228 (33.5)	151 (31.5)	77 (38.3)	
Days Before ICU (median [IQR])	1.00 [0.00, 4.00]	2.00 [0.00, 4.00]	0.00 [0.00, 8.00]	0.242
Days in ICU (median [IQR])	11.00 [7.00, 17.00]	11.00 [7.00, 16.00]	12.00 [8.00, 18.00]	0.028
Days in Hospital (median [IQR])	22.00 [15.00, 39.00]	20.00 [14.00, 34.00]	29.00 [19.00, 47.00]	<0.001
CCI (median [IQR])	1.00 [0.00, 2.00]	0.00 [0.00, 1.00]	1.00 [0.00, 2.00]	0.006
MFI (median [IQR])	1.00 [0.00, 2.00]	1.00 [0.00, 2.00]	1.00 [0.00, 2.00]	0.077
Hypertension (%)	380 (55.9)	260 (54.3)	120 (59.7)	0.224
Diabetes Mellitus (%)	216 (31.8)	157 (32.8)	59 (29.4)	0.433
Neoplasia (%)	41 (6.0)	22 (4.6)	19 (9.5)	0.024
Heart Failure (%)	31 (4.6)	14 (2.9)	17 (8.5)	0.003
COPD (%)	44 (6.5)	37 (7.7)	7 (3.5)	0.060
CKD (%)	27 (4.0)	15 (3.1)	12 (6.0)	0.130
Cirrhosis (%)	10 (1.5)	7 (1.5)	3 (1.5)	1.000
Septic Shock Day 1 (%)	200 (29.4)	174 (36.3)	26 (12.9)	<0.001
SAPS 3 Score (mean (SD))	48.47 (10.09)	49.33 (10.16)	46.43 (9.63)	0.001
Non-invasive Ventilation ≥ 1 hour (%)	252 (37.1)	210 (43.8)	42 (20.9)	<0.001
Mechanical Ventilation ≥ 1 hour (%)	83 (12.2)	62 (12.9)	21 (10.4)	0.436
Vasopressors ≥ 1 hour (%)	83 (12.2)	61 (12.7)	22 (10.9)	0.602
Non-invasive Ventilation (%)	353 (51.9)	297 (62.0)	56 (27.9)	<0.001
Mechanical Ventilation (%)	306 (45.0)	258 (53.9)	48 (23.9)	<0.001
Mechanical Ventilation Duration (mean (SD))	13.14 (13.54)	11.98 (11.75)	19.44 (19.77)	<0.001
Vasopressors (%)	256 (37.6)	213 (44.5)	43 (21.4)	<0.001
High-Flux Nasal Cannula (%)	240 (35.3)	238 (49.7)	2 (1.0)	<0.001
Tracheostomy (%)	61 (9.0)	55 (11.5)	6 (3.0)	0.001

	Overall	Private Hospital	Public Hospital	p
Venous Catheter (%)	544 (80.0)	428 (89.4)	116 (57.7)	<0.001
Arterial Catheter (%)	521 (76.6)	427 (89.1)	94 (46.8)	<0.001
Transfusion (%)	72 (10.6)	61 (12.7)	11 (5.5)	0.008
FFP (%)	9 (1.3)	8 (1.7)	1 (0.5)	0.394
ECMO (%)	18 (2.6)	18 (3.8)	0 (0.0)	0.012
Creatinine Day 1 (median [IQR])	1.00 [0.80, 1.23]	1.00 [0.80, 1.20]	0.98 [0.80, 1.32]	0.250
Overall AKI (%)	680 (100.0)	479 (100.0)	201 (100.0)	NaN
AKI				NaN
No AKI	0 (0.0)	0 (0.0)	0 (0.0)	
KDIGO 1	281 (41.3)	184 (38.4)	97 (48.3)	
KDIGO 2	203 (29.9)	149 (31.1)	54 (26.9)	
KDIGO 3	196 (28.8)	146 (30.5)	50 (24.9)	
Dialysis Modality (%)				<0.001
IHD	18 (20.7)	7 (9.6)	11 (78.6)	
SLED	42 (48.3)	39 (53.4)	3 (21.4)	
CRRT	27 (31.0)	27 (37.0)	0 (0.0)	
KRT (%)	87 (12.8)	73 (15.2)	14 (7.0)	0.005
Hemodialysis Duration (mean (SD))	17.76 (15.57)	17.79 (15.47)	17.62 (16.52)	0.970
