# Supplementary material for: Same disease, different outcomes: a retrospective cohort study of COVID-19–associated AKI across Brazil’s dual-tiered healthcare system
Source: J Bras Nefrol. 2025 Dec 12;48(2):e20250055. doi: 10.1590/2175-8239-JBN-2025-0055en (PMC12700444; doi:10.1590/2175-8239-JBN-2025-0055en)
Supplement: Table S4 [file 2175-8239-jbn-48-2-e20250055-suppl4.pdf]

Braz. J. Nephrol

https://doi.org/10.1590/2175-8239-JBN-2025-0055en

Supplementary Material to "Same Disease, Different Outcomes: A Retrospective Cohort Study of COVID-19–Associated AKI Across Brazil's Dual-Tiered Healthcare System"

TABLE S4 Difference in Survivors and Non-Survivors, stratified for Hospital Type.

		Private Hospital		Public Hospital		
	**Overall**	**Non-Survivor**	**Survivor**	**Non-Survivor**	**Survivor**	**p**
**Variables**	**2,333**	**405**	**887**	**486**	**555**	
**Demography**						
Age (mean (SD))		70.30 (13.39)	58.85 (15.84)	65.70 (14.53)	57.30 (16.26)	<0.001
Sex (%)	1419 (60.8)	269 (66.4)	582 (65.6)	266 (54.7)	302 (54.4)	<0.001
Ethnicity						<0.001
White	761 (32.6)	100 (24.7)	264 (29.8)	185 (38.1)	212 (38.2)	
Non-White	1572 (67.4)	305 (75.3)	623 (70.2)	301 (61.9)	343 (61.8)	
**Hospital Admission**						
Days Before ICU (median [IQR])	1.00 [0.00, 3.00]	2.00 [0.00, 4.00]	1.00 [0.00, 3.00]	0.00 [0.00, 3.00]	0.00 [0.00, 3.00]	<0.001
Days in ICU (median [IQR])	9.00 [5.00, 16.00]	14.00 [8.00, 23.00]	7.00 [3.00, 13.00]	11.00 [6.00, 19.00]	9.00 [5.00, 13.00]	<0.001
Days in Hospital (median [IQR])	17.00 [9.00, 29.00]	19.00 [12.00, 31.00]	15.00 [8.00, 25.00]	16.00 [9.00, 26.00]	19.00 [11.00, 34.00]	<0.001
**Comorbidities**						
CCI (median [IQR])	1.00 [0.00, 2.00]	1.00 [0.00, 2.00]	0.00 [0.00, 1.00]	1.00 [0.00, 2.00]	1.00 [0.00, 2.00]	<0.001
MFI (median [IQR])	1.00 [0.00, 2.00]	2.00 [1.00, 3.00]	1.00 [0.00, 2.00]	1.00 [0.00, 2.00]	1.00 [0.00, 2.00]	<0.001
Hypertension (%)	1363 (58.4)	284 (70.1)	470 (53.0)	294 (60.5)	315 (56.8)	<0.001
Diabetes Mellitus (%)	787 (33.7)	166 (41.0)	289 (32.6)	170 (35.0)	162 (29.2)	0.001
Neoplasia (%)	153 (6.6)	28 (6.9)	47 (5.3)	38 (7.8)	40 (7.2)	0.259
Heart Failure (%)	112 (4.8)	29 (7.2)	30 (3.4)	24 (4.9)	29 (5.2)	0.028
COPD (%)	142 (6.1)	54 (13.3)	62 (7.0)	14 (2.9)	12 (2.2)	<0.001
CKD (%)	89 (3.8)	25 (6.2)	23 (2.6)	18 (3.7)	23 (4.1)	0.019
Cirrhosis (%)	16 (0.7)	4 (1.0)	8 (0.9)	1 (0.2)	3 (0.5)	0.397
**Clinical Severity and Resource Use**						
Septic Shock Day 1 (%)	764 (32.7)	246 (60.7)	255 (28.7)	214 (44.0)	49 (8.8)	<0.001
SAPS 3 score (mean (SD))	49.57 (11.35)	58.64 (11.78)	48.03 (9.52)	51.47 (11.20)	43.75 (9.24)	<0.001
Non-invasive Ventilation ≥ 1 hour (%)	661 (28.3)	161 (39.8)	305 (34.4)	99 (20.4)	96 (17.3)	<0.001

		Private Hospital		Public Hospital		
	Overall	Non-Survivor	Survivor	Non-Survivor	Survivor	p
Mechanical Ventilation ≥ 1 hour (%)	418 (17.9)	115 (28.4)	80 (9.0)	181 (37.2)	42 (7.6)	<0.001
Vasopressors ≥ 1 hour (%)	404 (17.3)	110 (27.2)	84 (9.5)	173 (35.6)	37 (6.7)	<0.001
Non-invasive Ventilation (%)	927 (39.7)	223 (55.1)	434 (48.9)	135 (27.8)	135 (24.3)	<0.001
Mechanical Ventilation (%)	1103 (47.3)	362 (89.4)	315 (35.5)	342 (70.4)	84 (15.1)	<0.001
Mechanical Ventilation Duration (mean (SD))	14.11 (12.69)	15.93 (13.53)	11.69 (11.36)	14.07 (11.26)	15.73 (16.98)	<0.001
Vasopressors (%)	959 (41.1)	330 (81.5)	267 (30.1)	292 (60.1)	70 (12.6)	<0.001
High-Flux Nasal Cannula (%)	517 (22.2)	165 (40.7)	347 (39.1)	2 (0.4)	3 (0.5)	<0.001
Tracheostomy (%)	240 (10.3)	126 (31.1)	66 (7.4)	39 (8.0)	9 (1.6)	<0.001
Venous Catheter (%)	1638 (70.2)	393 (97.0)	620 (69.9)	407 (83.7)	218 (39.3)	<0.001
Arterial Catheter (%)	1521 (65.2)	383 (94.6)	620 (69.9)	344 (70.8)	174 (31.4)	<0.001
Transfusion (%)	281 (12.0)	135 (33.3)	80 (9.0)	45 (9.3)	21 (3.8)	<0.001
FFP (%)	29 (1.2)	17 (4.2)	8 (0.9)	3 (0.6)	1 (0.2)	<0.001
ECMO (%)	48 (2.1)	26 (6.4)	20 (2.3)	2 (0.4)	0 (0.0)	<0.001
**AKI characteristics and Recovery**						
Creatinine Day 1 (median [IQR])	1.04 [0.80, 1.40]	1.10 [0.90, 1.60]	1.00 [0.80, 1.20]	1.21 [0.85, 1.92]	0.99 [0.81, 1.30]	<0.001
Overall AKI (%)	1859 (79.7)	389 (96.0)	650 (73.3)	459 (94.4)	361 (65.0)	<0.001
AKI						<0.001
No AKI	474 (20.3)	16 (4.0)	237 (26.7)	27 (5.6)	194 (35.0)	
KDIGO 1	568 (24.3)	29 (7.2)	292 (32.9)	52 (10.7)	195 (35.1)	
KDIGO 2	388 (16.6)	49 (12.1)	186 (21.0)	69 (14.2)	84 (15.1)	
KDIGO 3	903 (38.7)	311 (76.8)	172 (19.4)	338 (69.5)	82 (14.8)	
Dialysis Modality (%)						<0.001
IHD	262 (50.1)	10 (5.4)	8 (9.9)	221 (95.7)	23 (85.2)	
PIKRT	161 (30.8)	105 (57.1)	42 (51.9)	10 (4.3)	4 (14.8)	
CKRT	100 (19.1)	69 (37.5)	31 (38.3)	0 (0.0)	0 (0.0)	
KRT (%)	523 (22.4)	184 (45.4)	81 (9.1)	231 (47.5)	27 (4.9)	<0.001
Hemodialysis Duration (mean (SD))	12.40 (13.11)	12.23 (12.18)	17.91 (15.76)	9.92 (11.39)	17.10 (15.92)	<0.001

CCI: Charlson Comorbidity Index; MFI: Modified Frailty Index; HAS: Systemic Arterial Hypertension; DM: Diabetes Mellitus; ICU: Intensive Care Unit; COPD: Chronic Obstructive Pulmonary Disease; CKD: Chronic Kidney Disease; SAPS 3: Simplified Acute Physiology Score 3; CF: Chronic Failure; TQT: Tracheostomy; FFP: Fresh Frozen Plasma; ECMO: Extracorporeal Membrane Oxygenation; AKI: Acute Kidney Injury; KRT: Kidney Replacement Therapy; HD: Hemodialysis.
