# Supplementary material for: Same disease, different outcomes: a retrospective cohort study of COVID-19–associated AKI across Brazil’s dual-tiered healthcare system
Source: J Bras Nefrol. 2025 Dec 12;48(2):e20250055. doi: 10.1590/2175-8239-JBN-2025-0055en (PMC12700444; doi:10.1590/2175-8239-JBN-2025-0055en)
Supplement: Translation

# Translation: Mesma doença, desfechos diferentes: um estudo de coorte retrospectivo da IRA associada à COVID-19 no sistema público e privado de saúde do Brasil

**DOI:** 10.1590/2175-8239-JBN-2025-0055pt

**Authors:** Natália Piazzi de Faria, José Hermógenes Rocco Suassuna, Carlos Augusto Moreira de Sousa, Sergio Miranda Freire, Conrado Lysandro R. Gomes

**Affiliations:** 1 Universidade do Estado do Rio de Janeiro, Hospital Universitário Pedro Ernesto, Faculdade de Ciências Médicas, Unidade Docente Assistencial de Nefrologia, Rio de Janeiro, RJ, Brasil.; 2 Kidney Assistance Ltda., Rio de Janeiro, RJ, Brasil.; 3 Complexo Médico Américas, Rio de Janeiro, RJ, Brasil.; 4 Fundação Oswaldo Cruz, Instituto Nacional de Saúde da Mulher, da Criança e do Adolescente Fernandes Figueira, Rio de Janeiro, RJ, Brasil.; 5 Universidade do Estado do Rio de Janeiro, Faculdade de Ciências Médicas, Departamento de Informação, Tecnologias e Educação em Saúde, Rio de Janeiro, RJ, Brasil.

**Keywords:** Injúria Renal Aguda, COVID-19, Disparidades em Assistência à Saúde, Mortalidade, Alocação de Recursos

## Abstract

**Introdução:**

A injúria renal aguda (IRA) é uma complicação crítica da COVID-19, e diferenças nos desfechos entre sistemas público e privado de saúde permanecem pouco exploradas no Brasil. Este estudo compara a epidemiologia da IRA em pacientes com COVID-19 em dois hospitais brasileiros - público e privado.

**Métodos:**

Estudo de coorte retrospectivo analisando 2.333 pacientes com COVID-19 confirmada por RT-PCR, na UTI (1.041 público; 1.292 privado; março/2020–abril/2022). IRA e recuperação seguiram critérios KDIGO e ADQI. Utilizou-se regressão logística multivariada e modelos de Cox para avaliar preditores de incidência e mortalidade por IRA. Para considerar risco competitivo de óbito intra-hospitalar, utilizou-se um modelo *Fine-Gray* para avaliar recuperação renal.

**Resultados:**

A incidência de IRA foi elevada nos dois contextos (privado, 80,4%; público, 78,8%). Apesar de características basais comparáveis, pacientes do hospital público apresentaram taxas de mortalidade significativamente superiores (46,7% vs. 31,3%; p < 0,001). Após ajuste para fatores de confusão, a admissão no hospital público permaneceu um preditor independente da incidência de IRA (OR 1,279; IC95%: 1,012–1,620) e mortalidade (HR 1,675; IC95%: 1,435–1,956). Embora taxas brutas de recuperação parecessem maiores nos hospitais públicos, a análise de risco competitivo revelou probabilidade de recuperação significativamente menor (SHR 0,650; IC95% 0,554–0,762).

**Discussão:**

Apesar da incidência semelhante de IRA, pacientes do hospital público apresentaram maior mortalidade e menor probabilidade de recuperação renal, provavelmente refletindo diferenças na disponibilidade de recursos. Esses achados ressaltam a necessidade de abordar a relação custo-efetividade e a equidade entre setores público e privado dos sistemas de saúde.

## Introdução

A injúria renal aguda (IRA) é uma complicação comum e grave da doença do coronavírus 2019 (COVID-19) e está associada a desfechos desfavoráveis tanto em curto quanto em longo prazo^1,2,3^. Desde o início da pandemia, um conjunto substancial de pesquisas destacou a complexidade e a elevada taxa de mortalidade da IRA em pacientes com COVID-19^4,5,6,7,8,9,10^.

O ônus global da COVID-19 e seu grave impacto na função renal aumentaram as disparidades no acesso aos cuidados em saúde e nos desfechos, particularmente em regiões com desigualdades socioeconômicas. Um estudo prospectivo sobre a incidência de IRA em 49 países revelou taxas mais elevadas de incidência e mortalidade em contextos de baixa e média-baixa renda, destacando as graves consequências do acesso limitado aos recursos de saúde^11^. De forma semelhante, uma revisão sistemática demonstrou que pacientes provenientes de contextos socioeconômicos menos favorecidos apresentaram piores desfechos, em decorrência do acesso restrito a recursos de cuidados intensivos^12^, os quais são essenciais para o manejo da IRA relacionada à COVID-19.

Apesar de extensas pesquisas, o impacto das disparidades socioeconômicas sobre a IRA no sistema de saúde dual público-privado do Brasil permanece incerto. Durante a pandemia, os dados indicaram que as regiões de menor nível socioeconômico e os hospitais públicos do país apresentaram pior desempenho, evidenciando as desigualdades no atendimento^13,14^. No entanto, não existem dados comparativos sobre as características e os desfechos de hospitais privados e públicos. Embora evidências anteriores sugiram a ausência de diferenças significativas nos desfechos gerais de IRA entre hospitais públicos e privados no Brasil^15^, a COVID-19 impôs desafios substanciais, com casos mais graves e maior demanda por cuidados intensivos^4^. Essas questões destacam a necessidade de uma compreensão mais aprofundada sobre como as disparidades de recursos impactam os desfechos, bem como de intervenções eficazes para abordar essas lacunas.

Este estudo teve como objetivo comparar a incidência, a recuperação e a mortalidade por IRA em pacientes com COVID-19 internados nas unidades de terapia intensiva (UTIs) de um hospital público e um hospital privado no Brasil, com foco em como as diferenças nos recursos de saúde entre esses dois contextos influenciam os desfechos.

## Métodos

### Desenho do Estudo

Este estudo de coorte retrospectivo incluiu pacientes com COVID-19 internados em UTIs de um hospital público (Hospital Universitário Pedro Ernesto/UERJ) e de um hospital privado (Complexo Médico Américas) localizados no Rio de Janeiro, Brasil, entre março de 2020 e abril de 2022. Por se tratar de um estudo observacional que utilizou dados anonimozados, os Comitês de Ética das instituições participantes aprovaram o estudo (número de aprovação 85672724.8.0000.5533) e dispensaram a exigência de consentimento informado. O estudo seguiu o protocolo STROBE (Material Suplementar).

### População do Estudo e Definições de IRA

Foram incluídos todos os pacientes com idade ≥ 18 anos que foram admitidos nas UTIs durante o período do estudo. Os casos de COVID-19 foram definidos como aqueles com resultado positivo no teste RT-PCR. Definiu-se IRA com base nos critérios KDIGO (*Kidney Disease Global Outcomes*) (Tabela S1)^16^. Considerou-se como creatinina basal o menor valor registrado durante a internação^4,6,16,17,18^. O débito urinário não foi registrado no sistema *Epimed Monitor* (ver abaixo) e, portanto, não foi utilizado para a classificação de IRA.

Foram excluídos os pacientes com doença renal crônica (DRC) avançada submetidos à terapia renal substitutiva (TRS) e aqueles com menos de duas medições de creatinina. Para pacientes com múltiplas admissões, foi considerada apenas a primeira hospitalização.

A recuperação renal e a doença renal aguda (DRA) – definida como IRA não resolvida com duração superior a sete dias – foram classificadas de acordo com os critérios da *Acute Dialysis Quality Initiative* (ADQI)^18^. A recuperação renal bruta foi definida como um nível final de creatinina sérica inferior a 25% acima do valor basal, em conformidade com os critérios propostos por Pannu et al.^19^. A recuperação foi classificada com base no intervalo de tempo: precoce (<48 h), persistente (48 h a 7 dias) e tardia (DRA, > 7 dias)^18,20^. Para pacientes com necessidade de TRS, a interrupção da diálise durante o acompanhamento indicou recuperação. Para os sobreviventes que não apresentaram recuperação em sete dias, foi aplicada a classificação ADQI para DRA, incluindo os estágios 0C e 1–3, de acordo com as definições da ADQI (Tabela S2)^18^. A taxa de filtração glomerular estimada foi calculada utilizando a equação CKD-EPI 2021, com base em idade, sexo e creatinina basal.

### Tamanho da Amostra, Coleta de Dados e Fontes

Entre março de 2020 e abril de 2022, 9.848 pacientes foram admitidos nas UTIs dos hospitais participantes. Após a exclusão, a coorte final foi composta por 2.333 pacientes (Figura 1). Os dados foram coletados prospectivamente na admissão à UTI. As informações do estudo foram obtidas a partir do sistema *Epimed Monitor* (*Epimed Solutions*, Rio de Janeiro, Brasil), um sistema estruturado de registros médicos eletrônicos utilizado por gestores de casos de UTIs^21^ para coleta e análise de dados de pacientes de UTI e enfermarias. Após o registro, os sujeitos foram acompanhados prospectivamente até a alta ou óbito hospitalar. O banco de dados foi anonimizado.

**Figura 1.** Fluxograma do estudo demonstrando a distribuição dos pacientes com base no tipo de hospital e na ocorrência de IRA (injúria renal aguda) na UTI (unidade de terapia intensiva). a. Refere-se a 1.011 pacientes com IRA que sobreviveram. b. Refere-se aos 87 pacientes que foram submetidos à TRS e sobreviveram. DRET – doença renal em estágio terminal, TRS – terapia renal substitutiva, CrS – creatinina sérica, DRA – doença renal aguda.

Os dados incluíram demografia, informações de UTI, comorbidades, Índice de Comorbidade de *Charlson* (ICC), Índice de Fragilidade Modificado (MFI), *Simplified Acute Physiology Score 3* (SAPS 3), choque séptico na admissão^22^, uso de recursos e terapias (vasopressores, ventilação não invasiva, ventilação mecânica [VM], oxigenação por membrana extracorpórea [ECMO]), e mortalidade na UTI/hospitalar. O registro dos dados foi realizado com marcação temporal (*timestamped*) para garantir a precisão da documentação. Revisamos manualmente os prontuários eletrônicos para verificar a completude das variáveis com dados ausentes. No entanto, excluímos a variável raça da análise multivariada, uma vez que aproximadamente metade dos prontuários apresentava dados ausentes. Da mesma forma, o índice de massa corporal (IMC) foi excluído devido à grande proporção de valores faltantes, visto que os dados antropométricos não foram coletados sistematicamente durante o período da pandemia.

### Desfechos Relevantes

Os principais desfechos comparados entre os hospitais público e privado incluíram a incidência e os fatores de risco para IRA, estratificados por estágios do KDIGO, a recuperação renal em pacientes com IRA, a necessidade de TRS e a mortalidade hospitalar em 30 dias.

### Análise Estatística

Os dados foram analisados utilizando o software estatístico R (versão 4.0.2). As variáveis contínuas são apresentadas como medianas com intervalos interquartis (IIQ) e comparadas por meio do teste U de Mann-Whitney. As variáveis categóricas são expressas como frequências e porcentagens e comparadas com o teste qui-quadrado. A significância estatística foi definida como p < 0,05.

Modelos de regressão logística univariada e multivariada foram utilizados para identificar preditores de incidência e recuperação de IRA. As covariáveis incluíram características demográficas e clínicas, como idade, sexo, ICC, MFI, hipertensão arterial (HA), diabetes mellitus (DM), DRC, obesidade, doença pulmonar obstrutiva crônica (DPOC), câncer, escore SAPS 3, VM, uso de vasopressores na admissão, gravidade da IRA (KDIGO 1–3) e tipo de hospital (público ou privado).

A multicolinearidade foi avaliada por meio do fator de inflação da variância (VIF), e as variáveis com alta colinearidade foram excluídas. Considerando que pacientes com maior tempo de internação e maior gravidade da doença são frequentemente transferidos para hospitais públicos, exploramos o potencial viés de seleção ao incluir um termo de interação (tipo de hospital × *status* de transferência) no modelo de regressão. Foram calculados *odds ratios* (OR) ajustados e intervalos de confiança (IC) de 95%. A seleção do modelo foi baseada no Critério de Informação de Akaike (AIC) e na área sob a curva (AUC), e o ajuste do modelo foi avaliado utilizando o teste de *Hosmer-Lemeshow*.

Uma vez que a mortalidade constitui um evento competitivo para a recuperação da IRA, foi aplicado um modelo de risco competitivo de *Fine-Gray* utilizando o pacote *cmprsk* no software R. A variável tempo até o evento foi o tempo de permanência hospitalar até a recuperação da IRA, sendo o óbito tratado como um evento competitivo. Os pacientes que permaneceram hospitalizados sem recuperação ou óbito foram censurados. Os preditores incluíram tipo de hospital, VM e gravidade da IRA (KDIGO estágios 1–3, modelados como variáveis ordinais). Foram relatadas *hazards ratio* de subdistribuição (SHR) com intervalos de confiança (IC) de 95%, em que valores de SHR > 1 indicaram maior probabilidade de recuperação.

Curvas de sobrevida de *Kaplan-Meier* foram utilizadas para estimar a mortalidade em 30 dias por tipo de hospital e as diferenças foram avaliadas utilizando o teste de log-rank. O evento primário foi a mortalidade intra-hospitalar, com acompanhamento iniciado na admissão à UTI e encerrado com óbito do paciente ou censura. Os pacientes que se recuperaram ou desenvolveram DRA foram censurados. Modelos univariados de riscos proporcionais de Cox foram empregados para identificar preditores de sobrevida, seguidos por um modelo multivariado de Cox para variáveis com p < 0,1 na análise univariada. A multicolinearidade elevada foi tratada por meio do VIF. A seleção retrógrada (*backward selection*) com base no AIC foi aplicada para refinar o modelo final. O modelo de Cox final incluiu tipo de hospital, IRA grave (KDIGO estágios 2/3), VM na admissão e idade, que foi estratificada por *status* de transferência, a fim de considerar possíveis vieses relacionados à via de admissão. O desempenho do modelo foi avaliado utilizando o teste de razão de verossimilhança e o índice de concordância. Os resultados foram expressos como *hazard ratios* (HR) com IC de 95%.

## Resultados

Um total de 9.848 pacientes foram admitidos nas UTIs dos hospitais participantes, com uma coorte final de 2.333 pacientes com COVID-19, que apresentaram resultados positivos no RT-PCR, sendo analisados após exclusão. Desses, 1.292 (55,4%) foram admitidos em hospital privado e 1.041 (44,6%) em hospital público. A Figura 1 mostra o fluxograma do estudo, incluindo incidência de IRA, padrões de recuperação e mortalidade. A incidência de IRA foi elevada, afetando 79,7% dos pacientes, sem diferença significativa entre os hospitais privado e público (80,4% vs. 78,8%; p = 0,315).

### Dados Demográficos, Hospitalização, Comorbididades e Gravidade Clínica

A idade mediana foi de 64 anos, sem diferença significativa entre os hospitais (65 vs. 63 anos; p = 0,082). A porcentagem de pacientes brancos foi mais elevada no hospital privado em comparação ao público (74,4% vs. 42,0%; p < 0,001) (Tabela 1). A mediana de permanência na UTI foi maior no hospital público (10 vs. nove dias; p = 0,002), porém a permanência hospitalar total não apresentou diferença significativa (p = 0,073). Os perfis de comorbidade foram semelhantes entre os grupos, com uma mediana mais elevada do ICC no hospital público (p = 0,010). Os pacientes do hospital privado apresentaram escores SAPS-3 mais elevados (mediana, 49 vs. 46; p < 0,001) e uma maior incidência de choque séptico na admissão (38,8% vs. 25,3%; p < 0,001). O uso de VM (52,3% vs. 40,9%; p < 0,001), vasopressores (46,2% vs. 34,8%; p < 0,001) e ventilação não invasiva (incluindo cânula nasal de alto fluxo) (50,9% vs. 25,9%; p < 0,001) foram mais comuns no hospital privado. A realização de cateterização arterial, transfusão sanguínea e oxigenação por membrana extracorpórea (ECMO) também foi maior no grupo do hospital privado (todos p < 0,001) (Tabela 1).

**Tabela 1:** Dados demogrÁficos, admissÃo hospitalar, comorbidades e gravidade clÍnica estratificados por tipo de hospital

	Total	Privado	Público	Valor de p
	2333 (100%)	1292 (55,4%)	1041 (44,6%)	
**Dados demográficos**				
Idade (mediana [IIQ])	64,00 [51,00; 73,00]	65,00 [51,00; 74,00]	63,00 [52,00; 72,00]	0,082
Raça (%)^a^				<0,001
Branca	761 (53,1)	364 (74,4)	397 (42,0)	<0,001
Não-branca	674 (46,9)	125 (25,6)	549 (58,0)	
Sexo masculino (%)	1419 (60,8)	851 (65,9)	568 (54,6)	<0,001
**Dados de Hospitalização**				
Dias pré-UTI (mediana [IIQ])	1,00 [0,00; 3,00]	1,00 [0,00; 3,00]	0;00 [0;00; 3,00]	<0,001
Permanência na UTI, dias (mediana [IIQ])	9,00 [5,00; 16,00]	9,00 [4,00; 16,00]	10,00 [6,00; 16,00]	0,002
Permanência hospitalar, dias (mediana [IIQ])	17,00 [9,00; 29,00]	16,00 [9,00; 27,00]	17,00 [10,00; 30,00]	0,073
Tipo de admissão hospitalar (%)				0,034
Clínica	2311 (99,1)	1282 (99,2)	1029 (98,8)	
Cirurgia eletiva	18 (0,8)	6 (0,5)	12 (1,2)	
Cirurgia de emergência	4 (0,2)	4 (0,3)	0 (0,0)	
**Comorbidades**				
Índice de Charlson (mediana [IIQ])	1,00 [0,00; 1,00]	0,00 [0,00; 1,00]	1,00 [0,00; 2,00]	0,010
MFI (mediana [IIQ])	1,00 [0,00; 2,00]	1,00 [0,00; 2,00]	1,00 [0,00; 2,00]	0,090
Hipertensão arterial sistêmica (%)	1363 (58,4)	754 (58,4)	609 (58,5)	0,978
Diabetes mellitus (%)	787 (33,7)	455 (35,2)	332 (31,9)	0,100
Insuficiência cardíaca congestiva (%)	112 (4,8)	59 (4,6)	53 (5,1)	0,623
Neoplasia (%)	153 (6,6)	75 (5,8)	78 (7,5)	0,120
Doença pulmonar obstrutiva crônica (%)	142 (6,1)	116 (9,0)	26 (2,5)	<0,001
Doença renal crônica (%)	89 (3,8)	48 (3,7)	41 (3,9)	0,864
Obesidade (%)	103 (4,4)	44 (3,4)	59 (5,7)	0,011
**Gravidade Clínica e Utilização de Recursos**				
Choque séptico na admissão (%)	764 (32,7)	501 (38,8)	263 (25,3)	<0,001
SAPS-3, pontos (mediana [IIQ])	48,00 [42,00; 55,00]	49,00 [43,00; 57,00]	46,00 [40,00; 54,00]	<0,001
VM na admissão (%)	418 (17,9)	195 (15,1)	223 (21,4)	<0,001
VM (%)	1103 (47,3)	676 (52,3)	426 (40,9)	<0,001
Duração da VM (mediana [IIQ])	10,00 [6,00; 19,00]	9,00 [6,00; 19,00]	11,00 [6,00; 19,00]	0,357
Vasopressores na admissão (%)	404 (17,3)	194 (15)	210 (20,2)	<0,001
Vasopressores (%)	959 (41,1)	597 (46,2)	362 (34,8)	<0,001
Ventilação não invasiva (%)	927 (39,7)	657 (50,9)	270 (25,9)	<0,001
Traqueostomia (%)	240 (10,3)	192 (14,9)	48 (4,6)	<0,001
Cateter arterial (%)	1521 (65,2)	1003 (77,6)	518 (49,8)	<0,001
Marcapasso temporário (%)	8 (0,3)	2 (0,2)	6 (0,6)	0,169
Nutrição parenteral total (%)	12 (0,5)	10 (0,8)	2 (0,2)	0,097
Transfusão sanguínea (%)	281 (12,0)	215 (16,6)	66 (6,3)	<0,001
Oxigenação extracorpórea (%)	48 (2,1)	46 (3,6)	2 (0,2)	<0,001

Abreviaturas – IIQ (Intervalo Interquartil); UTI (Unidade de Terapia Intensiva); MFI (Índice de Fragilidade Modificado), SAPS-3 (Simplified Acute Physiology Score 3); VM (Ventilação Mecânica).

Notas – a. Os dados referem-se a 1.435 pacientes com informações disponíveis sobre raça.

### Características da IRA E Preditores Clínicos

Ambos os tipos de hospital apresentaram distribuições semelhantes dos estágios KDIGO, sendo a maioria dos casos classificados como KDIGO 3 (37,4% no hospital privado vs. 40,3% no hospital público; p = 0,086) (Tabela 2). A TRS foi mais comum no hospital público (24,8% vs. 20,5%; p = 0,016). O uso das modalidades de TRS variou significativamente: a hemodiálise intermitente (HDI) foi mais frequente no hospital público (94,6%), enquanto a terapia renal substitutiva intermitente prolongada (TRSIP) e a terapia renal substitutiva contínua (TRSC) foram mais comuns no hospital privado (55,5% e 37,7%, respectivamente; p < 0,001). A duração mediana da TRS foi mais longa no hospital privado (11,0 vs. 7,0 dias; p < 0,001) (Tabela 2).

**Tabela 2:** Características da IRA e recuperação entre os tipos de hospita

	Total	Privado	Público	Valor de p
	2333 (100%)	1292 (55,4%)	1041 (44,6%)	
**Características da IRA**
Creatinina D1 (mediana [IIQ])	1,04 [0,80; 1,40]	1,00 [0,88; 1,28]	1,20 [0,90; 1,70]	<0,001
TFGe, D1 (mediana [IIQ])	73,6 [49,7; 95,2]	81,2 [60,0; 78,1]	60 [37,4; 84,8]	<0,05
IRA (%)	1859 (79,7)	1039 (80,4)	820 (78,8)	0,315
Injúria renal aguda, estágios (%)				0,086
Sem IRA	474 (20,3)	253 (19,6)	221 (21,2)	
KDIGO 1	568 (24,3)	321 (24,8)	247 (23,7)	
KDIGO 2	388 (16,6)	235 (18,2)	153 (14,7)	
KDIGO 3	903 (38,7)	483 (37,4)	420 (40,3)	
TRS (%)	523 (22,4)	265 (20,5)	258 (24,8)	0,016
Modalidade de TRS				<0,001
HDI	262 (50,1)	18 (6,8)	244 (94,6)	
TRSIP	161 (30,8)	147 (55,5)	14 (5,4)	
TRSC	100 (19,1)	100 (37,7)	0 (0)	
Duração mediana da TRS (mediana [IIQ])	8,00 [3,00; 17,00]	11,00 [4,00; 20,00]	7,00 [3,00; 14,00]	<0,001
**Recuperação (geral e em sobreviventes com IRA)**				
Recuperados, total (%)^a^	449 (24,2)	224 (21,6)	225 (27,4)	<0,01
Classificação da recuperação (%)^a^				<0,05
Não recuperados	1410 (75,8)	815 (78,4)	595 (72,6)	
Recuperação precoce (< 2 dias)	294 (15,8)	146 (14,1)	148 (18,0)	
IRA persistente (2 a 7 dias)	85 (4,6)	45 (4,3)	40 (4,9)	
Recuperação tardia da IRA (> 7 dias)	70 (3,8)	33 (3,2)	37 (4,5)	
Recuperados, sobreviventes (%)^b^	331 (32,7)	171 (26,3)	160 (44,3)	<0,001
Classificação da recuperação (%)^b^				<0,001
Não recuperados	680 (67,3)	479 (73,7)	201 (55,7)	
Recuperação precoce (< 2 dias)	230 (22,7)	119 (18,3)	111 (30,7)	
IRA persistente (2 a 7 dias)	57 (5,6)	32 (4,9)	20 (3,1)	
Recuperação tardia da IRA (> 7 dias)	44 (4,4)	20 (4,9)	24 (6,6)	
Recuperado após TRS^c^	87 (80,6)	73 (90,1)	14 (51,9)	<0,001
**Não recuperados**
DRA (%)^d^	680 (67,3)	479 (73,7)	201 (55,7)	<0,01
KDIGO DRA 0C	302 (44,4)	196 (40,9)	106 (53,7)	
KDIGO DRA 1	239 (35,1)	170 (35,5)	69 (34,3)	
KDIGO DRA 2	98 (14,4)	79 (16,5)	19 (9,5)	
KDIGO DRA 3	41 (6,1)	34 (7,1)	7 (3,5)	
Mortalidade na IRA (%)	848 (45,6)	389 (37,4)	459 (56)	<0,001
Mortalidade, geral (%)	891 (38,2)	405 (31,3)	486 (46,7)	<0,001

Abreviaturas – IIQ (Intervalo Interquartil); TFGe (Taxa de Filtração Glomerular Estimada); IRA (Injúria Renal Aguda); KDIGO (*Kidney Disease Improving Global Outcomes*); TRS (Terapia Renal Substitutiva), HDI (Hemodiálise Intermitente), TRSIP (Terapia Renal Substitutiva Intermitente Prolongada), TRSC (Terapia Renal Substitutiva Contínua) e DRA (Doença Renal Aguda).

Notas – a. Refere-se a todos os 1.859 pacientes com IRA, considerando o óbito como não recuperação.

b. Refere-se aos 1.011 pacientes com IRA que sobreviveram.

c. Refere-se aos 108 pacientes que foram submetidos à TRS e sobreviveram.

d. Refere-se aos 680 pacientes sobreviventes que não se recuperaram.

A análise de regressão logística multivariada demonstrou que escores SAPS-3 elevados (*odds ratio* [OR]: 1,049; IC 95%: 1,035–1,065; p < 0,001), VM (OR 9,721; IC 95%: 5,978–16,446; p < 0,001), HA (OR 1,487; IC 95%: 1,175–1,883; p < 0,001) e DRC (OR 4,662; IC 95%: 1,853–15,670; p < 0,01) foram fatores de risco significativos para IRA. A admissão no hospital público foi associada a um discreto aumento na probabilidade de IRA (OR 1,279; IC 95%: 1,012–1,620; p < 0,05) (Tabela 3).

**Tabela 3:** RegressÃo LogÍstica uni e multivariada para incidÊncia e recuperaÇÃo de ira

	Univariada			Multivariada		
	OR	IC 95%	p	OR	IC 95%	p
**Regressão logística para injúria renal aguda^a^**						
Hipertensão Arterial	1,804	1,473–2,211	<0,001	1,487	1,175–1,883	<0,001
Doença Renal Crônica	5,630	2,334–18,499	<0,001	4,662	1,853–15,670	<0,01
Hospital Público	0,903	0,738–1,107	0,326	1,279	1,012–1,620	<0,05
Ventilação Mecânica	22,751	15,66–34,69	<0,001	9,721	5,978–16,446	<0,001
SAPS-3 (por ponto)	1,084	1,071–1,098	<0,001	1,049	1,035–1,065	<0,001
Vasopressores	18,973	12,74–29,65	<0,001	2,650	1,555–4,636	<0,001
**Regressão logística para recuperação da função renal^b^**				
IRA KDIGO 1		Referência			Referência	
IRA KDIGO 2	0,196	(0,146–0,261)	<0,001	0,292	(0,214–0,393)	<0,001
IRA KDIGO 3	0,065	(0,048–0,086)	<0,001	0,143	(0,102–0,198)	<0,001
Ventilação Mecânica	0,099	(0,077–0,126)	<0,001	0,258	(0,193–0,341)	<0,001
Hospital Público	1,162	(0,970–1,392)	0,104	1,087	(0,880–1,344)	0,438

Abreviaturas – TFGe (Taxa de Filtração Glomerular Estimada); IRA (Injúria Renal Aguda); KDIGO (*Kidney Disease Improving Global Outcomes*); SAPS-3 (*Simplified Acute Physiology Score 3*).

Notas – a. *Hosmer-Lemeshow*: valor p = 0,675; Área sob a curva: 0,835 (IC 95%: 0,817–0,853);

b. *Hosmer-Lemeshow*: valor p = 0,9338; Área sob a curva: 0,7599 (IC 95%: 0,7399–0,7800).

### Recuperação da IRA e Preditores

As taxas brutas de recuperação da IRA foram mais elevadas no grupo do hospital público (27,4% vs. 21,6%; p < 0,01; sobreviventes: 44,3% vs. 26,3%; p < 0,001). A recuperação após TRS foi mais prevalente no hospital privado (90,1% vs. 51,9%; p < 0,001) (Tabela 2).

Os pacientes do hospital privado que não se recuperaram eram, com maior frequência, do sexo masculino (66,4% vs. 53,2%; p = 0,002). Choque séptico e escores SAPS-3 mais elevados foram mais comuns entre os pacientes do hospital privado que não se recuperaram (36,3% vs. 12,9%; p < 0,001; SAPS-3: 49,33 vs. 46,43; p = 0,001). A VM (53,9% vs. 23,9%; p < 0,001) foi mais frequente em pacientes não recuperados no hospital privado (Tabela S3).

A regressão logística ajustada demonstrou que a admissão no hospital público esteve associada a uma maior probabilidade de recuperação da IRA, embora sem significância estatística (OR 1,087; IC 95%: 0,880–1,344; p = 0,104). A VM (OR 0,258; p < 0,001) e estágios mais avançados de IRA (KDIGO 2: OR 0,292; p < 0,001; KDIGO 3: OR 0,143; p < 0,001) reduziram significativamente a probabilidade de recuperação (Tabela 3).

Em contraste com os achados do modelo logístico, o modelo de *Fine-Gray* demonstrou que a admissão no hospital público esteve associada a uma probabilidade de recuperação significativamente menor (SHR 0,650; IC 95%: 0,554–0,762; p < 0,001). A VM (SHR 0,222; p < 0,001) e a IRA grave (SHR 0,338; p < 0,001 por estágio) foram fortes preditores negativos de recuperação (Tabela 4).

**Tabela 4:** Modelo de risco competitivo de *fine-gray* para recuperaÇÃo da IRA

	HR de subdistribuição	IC 95%	p
Tipo de hospital (público vs. privado)	0,650	(0,554–0,762)	<0,001
Ventilação Mecânica	0,222	(0,174–0,283)	<0,001
Injuria Renal Aguda (por estágio)	0,338	(0,292–0,391)	<0,001

Abreviaturas – HR: *Hazards ratio* (razão de riscos).

### Análise de Sobrevida e Regressão de COX

A taxa de mortalidade geral foi mais elevada no grupo do hospital público (total: 46,7% vs. 31,3%; em pacientes com IRA; 56% vs. 37,4%; p < 0,001) (Figura 2a). As curvas de sobrevida de *Kaplan-Meier* demonstraram menor taxa de sobrevida no hospital público (p < 0,001) (Figura 2b).

**Figura 2.** a) Gráfico de barras apresentando os desfechos de alta hospitalar (sobreviventes vs. não sobreviventes) estratificados por tipo de hospital e *status* de IRA. b) Curva de sobrevida de *Kaplan-Meier* comparando pacientes de UTI de hospitais públicos e privados. O teste de log-rank indicou uma diferença significativa na sobrevida entre os tipos de hospital (p < 0,001).

Os não sobreviventes no hospital privado eram mais velhos (idade média de 70,3 vs. 65,7 anos; p < 0,001) e com maior probabilidade de serem do sexo masculino (66,4% vs. 54,7%; p < 0,001). Também apresentaram taxas mais elevadas de comorbidades, como hipertensão (70,1% vs. 60,5%; p < 0,001) e DM (41,0% vs. 35,0%; p = 0,001). A gravidade clínica foi superior entre os não sobreviventes no hospital privado, conforme indicado por escores SAPS 3 mais elevados (média 58,6 vs. 51,5; p < 0,001), maior incidência de choque séptico na admissão (60,7% vs. 44,0%; p < 0,001), VM (89,4% vs. 70,4%; p < 0,001) e uso de vasopressores (81,5% vs. 60,1%; p < 0,001) (Tabela S4).

No modelo final de riscos proporcionais de Cox, estratificado por *status* de transferência, os preditores críticos de mortalidade intra-hospitalar incluíram idade avançada (HR 1,031; IC 95%: 1,026–1,036; p < 0,001), VM (HR 2,548; IC 95%: 2,110–3,076; p < 0,001), gravidade da IRA (HR 1,423; IC 95%: 1,145–1,767; p = 0,001) e admissão no hospital público (HR 1,675; IC 95%: 1,435–1,956; p < 0,001) (Tabela 5).

**Tabela 5:** Riscos proporcionais de cox univariados e multivariados para mortalidade intra-hospitalar

	Univariada			Multivariada		
	HR	IC 95%	p	HR	IC 95%	p
Idade	1,029	1,0232–1,035	<0,001	1,031	1,026–1,036	<0,001
IRA KDIGO 2/3	1,388	1,1174–1,725	0,003	1,423	1,145–1,767	0,001
Índice de comorbidade de *Charlson*	1,012	0,9718–1,054	0,568	–	–	–
Diabetes Mellitus	0,91	0,7718–1,074	0,266	–	–	–
Ventilação Mecânica	2,408	1,9657–2,949	<0,001	2,548	2,110–3,076	<0,001
Índice de Fragilidade Modificado	1,021	0,9546–1,092	0,542	–	–	–
Hospital Público	1,966	1,6994–2,275	<0,001	1,675	1,435–1,956	<0,001
Sepse na admissão	1,289	1,0943–1,519	0,002	–	–	–
SAPS-3 (por ponto)	1,004	0,9967–1,011	0,290	–	–	–

Abreviaturas – IRA (Injúria Renal Aguda), KDIGO (*Kidney Disease Improving Global Outcomes*), SAPS-3 (*Simplified Acute Physiology Score 3*). HR: *Hazards ratio* (razão de riscos).

## Discussão

Este estudo demonstra a existência de disparidades importantes nos desfechos da IRA associada à COVID-19 entre hospitais públicos e privados no Brasil. Apesar das taxas de incidência semelhantes de IRA em ambos os contextos hospitalares, nossos achados revelaram diferenças significativas na mortalidade, utilização de recursos e recuperação, as quais podem ser atribuídas a variações no acesso e na qualidade dos cuidados em saúde. Essas disparidades ocorreram em um contexto mais amplo de desafios sistêmicos enfrentados pelo sistema de saúde brasileiro durante a pandemia, incluindo dificuldades bem documentadas quanto à coordenação nacional e distribuição de recursos^23^.

De forma consistente com nossos resultados, estudos sobre IRA durante a pandemia demonstraram que pacientes com COVID-19 não apenas apresentam maior incidência de IRA, mas também formas mais graves, conforme evidenciado por taxas mais elevadas de IRA estágio 3 e maior necessidade de TRS. Além disso, esses pacientes demandam mais recursos de cuidados intensivos, como VM, e apresentam maior gravidade da doença^4,5,6,9,24^. Mesmo após o controle para variáveis-chave, a influência independente do *status* da COVID-19 na incidência de IRA^4^ sugere que o vírus contribui para um fenótipo mais grave de IRA, potencialmente por meio de efeitos virais diretos, respostas inflamatórias intensificadas ou complicações sistêmicas associadas^1,10,25^. Uma correlação forte e independente entre VM e incidência de IRA (OR 9,721; IC 95%: 5,978–16,446), além da necessidade de TRS, também foi observada em múltiplas coortes de COVID-19^5,9^, possivelmente indicando a complexa interação pulmão-rim na patogênese da IRA^1,10,25^. Embora as incidências brutas da doença não tenham diferido entre os tipos de hospital, a admissão no hospital público manteve-se como um fator independente para IRA (OR 1,279; IC 95%: 1,012–1,620), apesar de uma maior gravidade geral da doença no hospital privado, conforme já observado em coortes anteriores e possivelmente associado ao menor uso de recursos^11^.

Um dos principais achados deste estudo foi a maior taxa de mortalidade entre pacientes com IRA no hospital público em comparação ao hospital privado (46,7% vs. 31,3%, respectivamente). Esse contraste acentuado persistiu mesmo após o controle para variáveis de confusão, como idade, comorbidades e gravidade clínica, sugerindo que as diferenças nos desfechos não se deviam exclusivamente ao estado de saúde dos pacientes no momento da admissão. A admissão no hospital público foi identificada como um preditor independente de maior mortalidade, destacando o impacto das iniquidades do sistema de saúde - fato que já havia sido observado na mortalidade geral por COVID-19 no Brasil^13^. Esses achados também estão de acordo com relatos globais que destacam piores desfechos em contextos de saúde com recursos limitados, que podem ser exacerbados durante crises de saúde pública, como a pandemia da COVID-19^11^. Poucos estudos comparando hospitais públicos e privados no Brasil durante a COVID-19 relataram piores desfechos em hospitais públicos e mistos^13^. No entanto, tais estudos não avaliaram diferenças na incidência ou características da IRA entre hospitais. Dados epidemiológicos prévios no Brasil sugerem que o tratamento em hospitais públicos não influenciou a sobrevida na IRA com necessidade de diálise^15^. No entanto, considerando a gravidade geral da apresentação da COVID-19 e o aumento na demanda por recursos de UTI, os hospitais públicos tendem a apresentar um desempenho inferior, em razão de maiores restrições, quando comparados aos hospitais privados. Um estudo de coorte multinacional em diferentes contextos de renda demonstrou que a IRA em pacientes com COVID-19 em ambientes com poucos recursos apresentou as maiores taxas de mortalidade hospitalar. Essa associação persistiu mesmo após o ajuste para a gravidade da doença^11^. Portanto, a renda e a disponibilidade de recursos podem influenciar diretamente os desfechos de pacientes com COVID-19. Outro relato demonstrou que, durante o pico de gravidade da COVID-19, hospitais com recursos sobrecarregados, incluindo aqueles com alta taxa de ocupação nas UTIs, apresentaram taxas de mortalidade mais elevadas. Esses achados foram mais acentuados em regiões com recursos de saúde limitados^26^. Além disso, os hospitais públicos no Brasil frequentemente atendem uma proporção significativa da população de menor nível socioeconômico, podendo apresentar acesso mais tardio aos serviços de saúde, menos recursos e um cuidado pós-agudo menos abrangente em comparação aos hospitais privados^27^.

Em nosso estudo, a incidência aumentada de TRS e o uso frequente de HDI em hospitais públicos sugerem que essas instituições tiveram acesso limitado a modalidades mais avançadas ou contínuas de TRS, que são melhor toleradas em pacientes com instabilidade hemodinâmica e permitem um controle volêmico e suporte nutricional mais eficazes, ambos relevantes no cuidado de pacientes com COVID-19^2,25^. De forma semelhante a estudos nacionais que evidenciaram as iniquidades na assistência à saúde no Brasil^13,14^, nossos dados revelaram diferenças substanciais na disponibilidade e no uso de recursos avançados entre os tipos de hospital. Os pacientes do hospital privado foram mais frequentemente submetidos à ventilação não invasiva (incluindo cânula nasal de alto fluxo), ECMO, cateterismo arterial, transfusões sanguíneas e traqueostomia. Essas disparidades provavelmente refletem um melhor acesso à infraestrutura de cuidados intensivos e tecnologias especializadas em ambientes privados, o que pode explicar, em parte, as diferenças observadas nos desfechos de mortalidade e recuperação.

Devido ao elevado percentual de dados ausentes, a variável raça não pôde ser incluída em nossos modelos multivariados. Evidências provenientes de países de alta renda e do Brasil indicaram piores desfechos gerais em pacientes de ascendência africana^12,14^. A incidência de IRA também foi maior em pacientes de ascendência africana nos países de alta renda, mesmo após o ajuste para fatores socioeconômicos^4,8^. No entanto, apesar das conhecidas diferenças raciais na IRA, dados epidemiológicos abrangentes sobre a doença no Brasil não identificaram a raça como um fator significativo para a mortalidade por IRA^15^, e modelos preditivos de IRA e TRS no contexto da COVID-19 no país consistentemente não incluíram a raça como covariável^28^.

Um achado inicial contraintuitivo foi a maior recuperação renal aparente da IRA em hospitais públicos. Isso pode ser atribuído ao estágio mais grave da doença em hospitais privados, onde os pacientes necessitam de VM com mais frequência e apresentam escores de gravidade global mais elevados. No entanto, a regressão logística não considera eventos competitivos, particularmente a mortalidade, o que pode ter introduzido um viés de sobrevivência nas taxas de recuperação. Para abordar essa questão, aplicamos um modelo de risco competitivo de *Fine-Gray*, que confirmou que a admissão no hospital público esteve associada a uma probabilidade significativamente menor de recuperação renal (SHR 0,650; IC 95%: 0,554–0,762; p < 0,001). Esses achados indicam que as taxas de recuperação mais altas observadas em hospitais públicos por meio da regressão logística foram confundidas pelas taxas de mortalidade mais elevadas nesses pacientes, uma vez que muitos indivíduos gravemente enfermos não sobreviveram tempo suficiente para apresentar recuperação renal. A recuperação completa após a TRS foi mais prevalente em hospitais privados (90,1% vs. 51,9%; p < 0,001). Isso pode refletir diferenças na gravidade da doença que levou ao início da TRS, na disponibilidade de recursos, nas estratégias de tratamento e na presença de profissionais de saúde qualificados^13^. Os hospitais privados podem ter oferecido um cuidado mais abrangente para casos graves de IRA, incluindo o uso mais intenso de TRSC, o que pode ter resultado em melhor recuperação da função renal entre aqueles com necessidade de TRS^25^. No entanto, essa superioridade permanece discutível^29^. Esse padrão foi ainda mais reforçado pela acentuada disparidade na dependência de TRS entre os pacientes com DRA estágio 3, dos quais 48,1% nos hospitais públicos, em comparação a apenas 9,9% nos hospitais privados, permaneceram dependentes dessa terapia. Esses achados destacam a influência da disponibilidade de recursos e da qualidade do cuidado nas trajetórias de recuperação renal após IRA grave.

A elevada prevalência de DRA em sobreviventes da COVID-19 destaca as implicações renais de longo prazo da doença e a necessidade de estratégias de acompanhamento e manejo prolongados^3^. Fisher et al.^4^ descreveram que as taxas de recuperação foram mais baixas em pacientes com COVID-19 e IRA, com apenas 42,3% apresentando recuperação renal, em comparação a 68,5% em pacientes com IRA não relacionada à COVID. Dados recentes sugerem que muitos pacientes apresentam comprometimento renal persistente após a recuperação da COVID-19 aguda^30^. Essas observações destacam a necessidade de acompanhamento vigilante e intervenções terapêuticas precoces para mitigar o impacto de longo prazo da injúria renal em sobreviventes da COVID-19.

Este estudo apresenta diversos pontos fortes e limitações. Os pontos fortes incluem o grande número de pacientes, a coleta prospectiva de dados e a reflexão sobre os cuidados de mundo real. As limitações incluem o desenho retrospectivo e os potenciais vieses decorrentes da ausência de dados raciais, bem como dados antropométricos incompletos, o que impediu análises com base no IMC. Como em qualquer estudo observacional, não é possível descartar a existência de fatores de confusão residuais e não mensurados. A amostra do estudo foi proveniente de uma região geográfica específica, o que pode limitar a generalização para outras populações ou contextos. Outra limitação foi a ausência de acompanhamento de longo prazo dos sobreviventes. Um viés potencial foi o encaminhamento de pacientes provenientes de outras unidades públicas, muitos já admitidos com VM, e pacientes em estado crítico, o que pode ter influenciado a mortalidade no hospital público. No entanto, todos os modelos foram ajustados para o *status* de transferência, indicando que o encaminhamento tardio não afetou de forma significativa as disparidades observadas nos desfechos entre os tipos de hospital.

## Conclusões

Este estudo constatou que, apesar da incidência semelhante de IRA, os pacientes com COVID-19 admitidos no hospital público apresentaram mortalidade significativamente mais elevada, menor probabilidade de recuperação renal após consideração de riscos competitivos e acesso reduzido a tecnologias avançadas de suporte à vida, como TRSC e ECMO. Esses achados enfatizam a necessidade urgente de reavaliar a forma como os recursos de saúde são alocados em diferentes contextos hospitalares e de considerar a equidade no cuidado em sistemas de saúde duais nos países emergentes. O fortalecimento da capacidade de cuidados intensivos e a promoção de acesso equitativo a terapias avançadas permanecem essenciais para a melhoria dos desfechos em ambientes com recursos limitados.

## Data Availability

O conjunto de dados analisado durante o presente estudo está disponível mediante solicitação razoável ao autor correspondente.

## Material Suplementar

O seguinte material online está disponível para este artigo:

Tabela S1 Definições de injúria renal aguda com base nas definições do KDIGO.

Tabela S2 Definições de doença renal aguda de acordo com as diretrizes do ADQI.

Tabela S3 Diferenças entre pacientes não recuperados de acordo com o tipo de hospital.

Tabela S4 Diferenças entre sobreviventes e não sobreviventes, estratificadas por tipo de hospital.

Checklist das diretrizes STROBE.
